# Characteristic tetanus infection in disaster-affected areas: case study of the Yogyakarta earthquakes in Indonesia

**DOI:** 10.1186/1756-0500-2-34

**Published:** 2009-03-06

**Authors:** Agung Budi Sutiono, Andri Qiantori, Hirohiko Suwa, Toshizumi Ohta

**Affiliations:** 1 The University of Electro-Communications, Graduate School Information Systems, Graduate Department Social Intelligence and Informatics, 1-5-1 Chofugaoka, Chofu-shi, Tokyo, 182-8585 Japan; 2 Hasan Sadikin University Padjadjaran Hospital, Jl. Pasteur 38 Bandung, 40161 Indonesia

## Abstract

**Background:**

Tetanus is an infectious disease caused by the contamination of wounds from bacteria that live in soil. The tetanus mortality rate remains high in developing countries affected by natural disasters. Whether the socio-demography and geographical conditions may influence the tetanus treatment outcome on the earthquake situation in Yogyakarta, Indonesia has not been investigated.

**Findings:**

We present 26 tetanus patients who were admitted to eight hospitals following the earthquakes that occurred on May, 27, 2006, in Yogyakarta, Indonesia. The independent variables were age, gender, distance, admission, hospitalization, and type of hospital with the dependent variable surviving or perishing. Data were analyzed by logistic regression methods on SPSS 17.0. The distance from the patient's place of residence to the hospital were obtained and analyzed by using geospatial tools MapInfo 7.8 SCP and Global Mapper 7. Eight of the 26 patients were dead (30.8%) and statistical results showed that the distance (OR = 1.740, 95% CI = 1.068–2.835) and type of hospital (OR = 0.067, 95% CI = 0.001–3.520) were significant predictors of death.

**Conclusion:**

Our findings show that in order to reduce the mortality rates, performing triage systems based on the distance and type of hospital priority for internally displaced persons could be proposed as well as making provisions for the generally old population in order to prevent an outbreak of tetanus following earthquakes in Yogyakarta, Indonesia.

## Background

**Figure 1:**
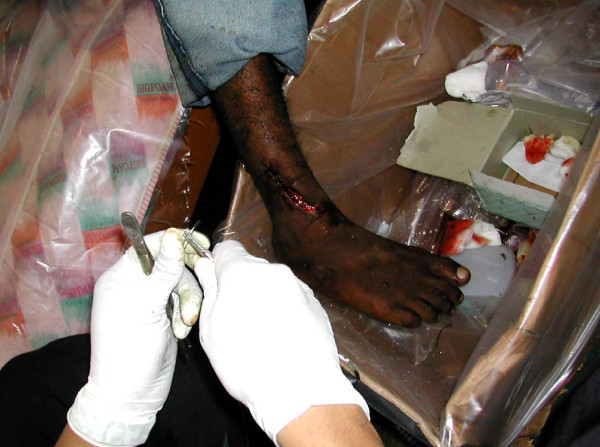
**Open wound on disaster is susceptible for Clostridium tetani**.

Tetanus is a devastating disease involving muscle spasms and autonomic instability and associated with a high incidence of mortality. Despite being easily preventable, with a highly effective vaccine, tetanus remains a significant source of morbidity and mortality worldwide [1,2]. The majority of tetanus cases occur in third-world countries. Tetanus is caused by *Clostridium Tetani *[3,4], a bacterium that infects open wounds commonly occurring in disaster-affected areas. Open wounds, such as lacerations, abrasions, and punctures, are a significant source of bacterial infection [3]. Earthquakes can strike quickly and without warning, forcing the evacuation of those who are injury-prone. On the other hand, increasing cases of tetanus observed in the elderly population are attributable to immune systems that decline with advancing age [5]. Elderly people, women and children are susceptible to injury, and consequently *Clostridium tetani *may enter the body through dirty open wounds (Figure 1). Occasionally, tetanus affects only the part of the body where the infection originates, but in almost all reported cases, the infection spreads to the entire body. The incubation period from the time of injury until the appearance of the first symptoms ranges from 2 to 50 days. Symptoms usually occur within 5 to 10 days. The early appearance of symptoms is associated with an increased chance of death and the overall mortality rate is approximately 10%–50% [6,7]. Whether the socio-demography and geographical conditions may influence the tetanus treatment outcome on the circumstances around earthquakes in Yogyakarta, Indonesia has not been investigated.

## Methods

In this study, we examined twenty six tetanus patients who had been admitted to eight hospitals as part of the post-disaster responses in Bantul County, Yogyakarta province, Indonesia, eight of whom were dead. On June 14, 2006 (19 days after the earthquakes) data were collected from Sardjito General Hospital, Wates Hospital, Muhammadiyah Yogyakarta Hospital, Muhammadiyah Bantul Hospital, Harjo Lukito Hospital, Ludira Husada Hospital, Panembahan Senopati Hospital, and Walubi Field Hospital (run by the Indonesian Buddhist Society) through the Bantul County local health office.

**Figure 2:**
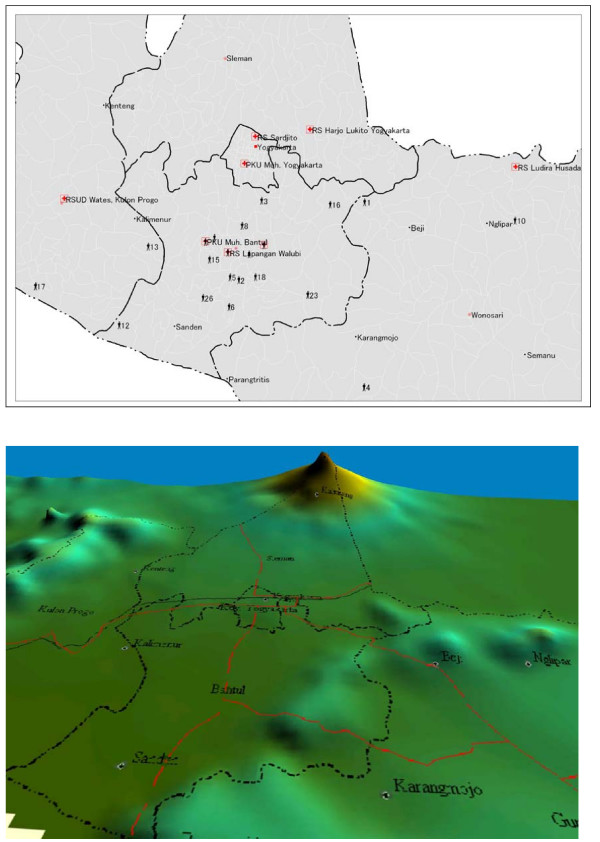
**Tetanus patient's origin, hospitals location and geographical 3D landscape on the earthquakes in Yogyakarta Indonesia**. Geospatial view by using MapInfo Professional 7.8. SCP. Geospatial view by using Global Mapper 7. road = red line. county border = dots and dashes.

Using the MapInfo professional 7.8 geospatial tool, the patient's location origin and the hospital's coordinates for latitude and longitude were used to determine the distance from the patient's position origin to the hospital. The hospital coordinates were obtained from Wikimapia: Indonesia/Yogyakarta , supported by Google Earth. The residences of the individual patients were geocoded using MapInfo Professional 7.8. based on their address. The distance was measured from the patient's origin to the hospital location by tracking the road map and geographical contours on the digital map via Global Mapper 7 (Figure 2). The distance was classified in 2 groups (under 15 km and 15 km or more). The distance classification was related to the response time from the patient's origin to the hospital, since the ambulance service in Yogyakarta was considered to be good.

Admission means the length in terms of the number of days from the initial injuries and open wound contaminated by *Chlostridium tetani *until they were admitted to hospital and was classified into below 7 days and 7 days or more. Thereafter the hospitalization referred to the length of stay in the hospital, which was also classified into below 7 days and 7 days or more.

The hospital type was classified based on the Ministry of Health of Indonesia (MOH) standard. There are 4 levels of hospital in Indonesia, specifically, types A, B, C and D (A representing the highest level and D the lowest). The hospital type is classified based on the standard of health services provided and the patient facilities for patients. The Sardjito Hospital is the top academic referral hospital in Yogyakarta and categorized as type B. Six of the remaining hospitals were categorized as type C, and one (Walubi field hospital) as type D. The characteristics of those eight hospitals were collected from the Indonesian hospital association, in the form of a data information website .

SPSS 17.0 was applied by using the binary logistic regression analyses method. Logistic regression is particularly relevant, since the survival output as the dependent variable is binary, with the output coded as 1 = death and 0 = survival. Socio-demographic and geographical information data were also employed as independent variables, namely in the form of age, gender, distance, admission, hospitalization, and type of hospital. Age was classified into two groups, those under 60 and those of 60 years or more respectively, since immune systems decline in individuals aged over 60 years [5]. There was also gender separation as well.

## Results

The characteristics of tetanus patients are shown in Table 1. The age classification of those 60 years or more included 15 patients (57.7%), ranging in age from 20 to 89 years. The total was predominantly male (n = 20, 76.9%), relative to female (n = 6, 23.1%). Distance was classified into two groups, with 69.2% of patients (n = 18) located under 15 km away and the remaining 30.8% patients (n = 8) located 15 km or more from their location to the hospital. In the early stages of tetanus, the majority of patients were admitted to the hospital for under 7 days (n = 17, 65.4%), with 9 patients (34.6%) admitted for 7 days or more. In most cases, the hospitalization periods were 7 days or more, for 69.2% n = 18. Thirteen patients (50%) were treated in the type B hospital, eleven patients (42.3%) in the type C hospital and two patients (7.7%) in the type D hospital.

**Table 1:** Characteristic 26 tetanus patients on the earthquakes in Yogyakarta Indonesia

**Variables**	**Category**	**Number of patients**	**Percentage**
Age (20–89 years old) Range 69	< 60 years old	11	42.3
	≥ 60 years old	15	57.7

Gender	Male	20	76.9
	Female	6	23.1

Distance	< 15 km	18	69.2
	≥ 15 km	8	30.8

Admissions	< 7 days	17	65.4
	≥ 7 days	9	34.6

Hospitalization	< 7 days	8	30.8
	≥ 7 days	18	69.2

Type hospital	Type B	13	50.0
	Type C	11	42.3
	Type D	2	7.7

Live-die	Live	18	69.2
	Die	8	30.8

**Figure 3:**
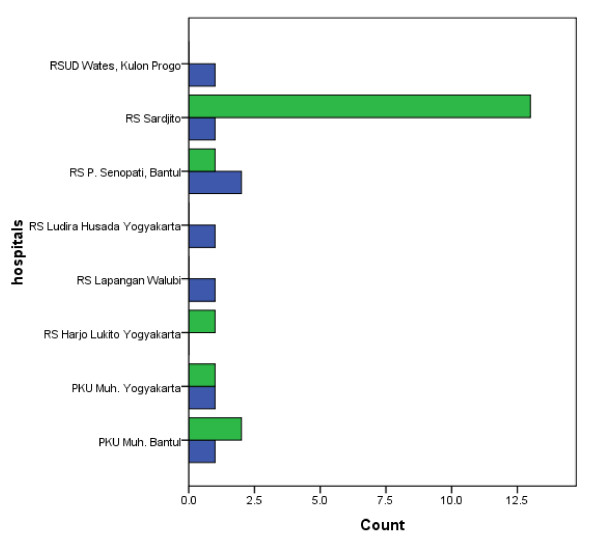
**Tetanus patient's distribution by hospitals on the earthquakes in Yogyakarta Indonesia**. Blue = die. Green = live.

The patient's distribution graph based on live and die in each hospital is described on figure 3. Most of patients were admitted to the Sardjito hospital. The death cases had been reported in Wates hospital, Sardjito hospital, Panembahan Senopati hospital, Ludira Husada hospital, Walubi field hospital, Muhammadiyah Yogyakarta hospital and Muhammadiyah Bantul hospital after admission and there was no dead case in Harjo Lukito hospital.

In Table 2, the mean age of patients who died was 74.62 ± 13.43 years old; a figure which differed significantly (p = 0.013, significant level p < 0.05) from the mean age of the surviving patients, namely 54.94 ± 18.72 years old. The other mean values for those patients dying also differ significantly to those surviving, namely in terms of distance 29.83 ± 6.74 Km (p < 0.0001), admission 18.12 ± 3.44 days (p < 0.0001), hospitalization 3.12 ± 1.64 days (p < 0.0001), and type of hospital 2.25 ± 0.46 (p < 0.0001).

**Table 2:** Mean value by death-live outcome of tetanus patients on the earthquakes in Yogyakarta Indonesia

**Variables**	**Patient outcome**	**Means**	**SD**	**P-value**
Age (years old)	Death	74.62	13.43	*0.013
	
	Live	54.94	18.72	

Distance (Km)	Death	29.83	6.74	** < 0.0001
	
	Live	6.50	2.26	

Admission (days)	Death	18.12	3.44	** < 0.0001
	
	Live	3.33	1.64	

Hospitalization (days)	Death	3.12	1.64	** < 0.0001
	
	Live	16.83	4.33	

Type hospital	Death	2.25	0.46	** < 0.0001
	
	Live	1.28	0.46	

*Significant at level p < 0.05 **Significant at level p < 0.01

Statistical analysis using the binary logistic regression method showed that the variables of distance and type of hospital were significant (at level P < 0.05) on P = 0.026 (OR = 1.740, 95% CI = 1.068–2.835) and P = 0.018 (OR = 0.067, 95% CI = 0.001–3.520) respectively. Meanwhile age, gender, admission and hospitalization were not statistically significant for tetanus cases during the earthquakes in Yogyakarta (Table 3).

**Table 3:** Multivariate analysis tetanus cases by logistic regression on the earthquakes in Yogyakarta Indonesia

**Variables**	**OR**	**95% Confidence Interval**		**P-value**
			
		**Lower Bound**	**Upper Bound**	
Age	0.995	0.866	1.142	0.939

Gender	0.422	0.002	93.369	0.754

Distance	1.740	1.068	2.835	*0.026

Admission	0.981	0.411	2.343	0.966

Hospitalization	1.316	0.284	6.091	0.726

Type hospital	0.067	0.001	3.520	*0.018

*Significant at level p < 0.05

The characteristic of hospital are different based on the type B, C and D in term of human resources and facilities. The Sardjito hospital is the biggest hospital with 631 beds, 24 general practitoners (GP), 198 medical spesialist, 564 residents, 799 nurses and many facilities such as X-ray, CT scan, USG, ECG, EEG, EMG, emergency room, operating room and ICU (intensive care unit) are also available in comparison to the other hospital .

## Discussion

Tetanus is an infectious disease caused by a toxin produced by *Clostridium tetani*, which is found in soil, human feces, and objects lying on the ground. Tetanus infections are common in disaster-affected areas. Tetanus cases were also reported by Australian humanitarian assistance 13 days after the tsunami of 26 December, 2006 [8]. Open wounds are the general entry points for bacteria. In developing countries, where there is normally a lack of facilities, tetanus can be fatal by paralyzing the breathing muscles, causing sudden death in disaster-affected areas. In this study, 26 tetanus cases after the Yogyakarta earthquakes were examined in order to establish the associated factors, with a view to minimizing mortality due to the disease. In 26 tetanus patients, 8 were dead and binary logistic regression analysis by SPSS 17.0 and GIS geospatial tools were applied to analyze the socio-demography and geographical information. We restricted the analysis to cover solely the disaster-affected areas, socio-demography and GIS.

The characteristic findings showed that most patients were male (76.9% n = 20), aged 60 years or more (57.7%, n = 15), while in a previous study, gender and age were applied as standard demographic variables. Following the tsunami in Banda Aceh, Jeremijenko *et al*. reported 106 tetanus cases, 79% of whom came in individuals over 25 years (median age of 40 years) and 62% of whom were male [9]. However, no tetanus studies were performed in relation to socio-demography and geographical variables, such as distance to the health facilities, admission, hospitalization and hospital type [9]. During natural disasters, a history of injury is recorded in more than 70% of patients presenting with tetanus [10]. The majority of patients are inadequately immunized against tetanus [11], particularly the elderly population. Tetanus toxoid immunization is believed to be nearly 100% effective in preventing tetanus infections [12,13]. Some individuals may be protected for life, but most people have antitoxin levels that approach the minimal protective level 10 years after the previous dose. Since patients were predominantly 60 years or more (57.7%), their immune systems were in decline and a booster was essential to prevent tetanus. The previous study in the United States reported 3.8% of tetanus cases treated by the disaster medical assistance team after earthquakes [14]. There was no report of deaths attributable to the treatment administered, including tetanus boosters [15]. Concerning gender, males seemed to be more active during the disaster, during which they had to evacuate their relatives, meaning they (76.9%) were more susceptible to infection than females (23.1%).

In our findings, the distance and type of hospital are significant risk factors when considering the survival of tetanus patients following an earthquake scenario. The greater the distance or time involved (OR = 1.740, 95% CI = 1.068–2.835), the greater the risk of mortality for tetanus cases. This is related to the ambulance service in Yogyakarta province, which has a good reputation. We assumed that average ambulance speed of 50 km/hour, the time required to transport patients from their residence to the nearest hospital within a radius of under 15 km is approximately 18 minutes. It is still reasonable to consider whether the paramedics should perform CPR (cardiopulmonary resuscitation) during the evacuation while the consideration to stop times taken to try CPR is as simple as an isolated time interval (around 30 minutes). Nevertheless the clinical judgment and respect for human dignity must also enter for this decision [16]. Meanwhile, upgrading the type (level) of hospital will significantly (OR = 0.067, 95% CI = 0.001–3.520) decrease the mortality risk for tetanus patients. The characteristics of the hospital differ completely depending on its type B, C or D in terms of human resources and facilities. Sardjito hospital is the biggest of those covered, with 631 beds, 24 general practitioners (GPs), 198 medical specialists, 564 residents, 799 nurses and many facilities such as X-rays, CT scans, USG, ECG, EEG, EMG, emergency room, operating room and ICU (intensive care unit) in comparison to the other hospitals .

Nevertheless successful treatment also depends on numerous other factors. Based on an analysis of tetanus cases after the Yogyakarta earthquakes, proposed referral systems for critically ill tetanus patients should be considered as well as the ambulance systems, which have been effective in Yogyakarta. GIS was significant in logistic regression, and will hence be useful for distance analysis on this study. We realize that analysis of other independent variables must be lacking due to the limited variables in this study. For those reasons we restricted this study to the circumstances of the earthquakes, socio-demography and geographical data setting.

## Conclusion

Our findings show that in order to reduce the mortality rates, performing triage systems based on the distance and type of hospital priority for internally displaced persons could be proposed as well as making provisions for the generally old population in order to prevent an outbreak of tetanus following earthquakes in Yogyakarta, Indonesia.

## Competing interests

The authors declare that they have no competing interests.

## Authors' contributions

AQ and HS assisted the statistical analyses and interpreting the data. TO has given a suggestion how to choose the methodology analysis and involved in revising the manuscript critically before submission. Author and co-authors have read and approved the manuscript.
