# Characteristic tetanus infection in disaster-affected areas: case study of the Yogyakarta earthquakes in Indonesia

**DOI:** 10.1186/1756-0500-3-8

**Published:** 2010-01-18

**Authors:** Agung Budi Sutiono, Andri Qiantori, Hirohiko Suwa, Toshizumi Ohta

**Affiliations:** 1 The University of Electro-Communications, Graduate School Information Systems, Graduate Department Social Intelligence and Informatics, 1-5-1 Chofugaoka, Chofu-shi, Tokyo, 182-8585 Japan; 2 Hasan Sadikin University Padjadjaran Hospital, Jl. Pasteur 38 Bandung, 40161 Indonesia

## Correction

After publication of this work [1], we noted that we inadvertently failed to include the new version of figure two that needs correction. The location of "RS Ludira Husada" is near Yogyakarta, not near "Nglipar". This change will not alter the statistical analysis and results since we used the corrected version of figure two (fig. 1 in this manuscript) for calculation. We profusely regret this mistake and offer our sincerest apologies and correct the figure two as below.
